# Trade-off in membrane distillation with monolithic omniphobic membranes

**DOI:** 10.1038/s41467-019-11209-6

**Published:** 2019-07-19

**Authors:** Wei Wang, Xuewei Du, Hamed Vahabi, Song Zhao, Yiming Yin, Arun K. Kota, Tiezheng Tong

**Affiliations:** 1 Department of Mechanical Engineering, Colorado State University, Fort Collins, CO 80523 USA; 2 Department of Civil and Environmental Engineering, Colorado State University, Fort Collins, CO 80523 USA; 3 School of Chemical Engineering and Technology, Tianjin Key Laboratory of Membrane Science and Desalination Technology, Tianjin University, Tianjin, 300072 China; 4 Department of Chemical and Biological Engineering, Colorado State University, Fort Collins, CO 80523 USA; 5 School of Biomedical Engineering, Colorado State University, Fort Collins, CO 80523 USA; 6 School of Advanced Materials Discovery, Colorado State University, Fort Collins, CO 80523 USA; 7 Department of Mechanical and Aerospace Engineering, North Carolina State University, Raleigh, NC 27695 USA

**Keywords:** Mechanical engineering, Wetting, Chemical engineering

## Abstract

Omniphobic membranes are attractive for membrane distillation (MD) because of their superior wetting resistance. However, a design framework for MD membrane remains incomplete, due to the complexity of omniphobic membrane fabrication and the lack of fundamental relationship between wetting resistance and water vapor permeability. Here we present a particle-free approach that enables rapid fabrication of monolithic omniphobic membranes for MD desalination. Our monolithic omniphobic membranes display excellent wetting resistance and water purification performance in MD desalination of hypersaline feedwater containing surfactants. We identify that a trade-off exists between wetting resistance and water vapor permeability of our monolithic MD membranes. Utilizing membranes with tunable wetting resistance and permeability, we elucidate the underlying mechanism of such trade-off. We envision that our fabrication method as well as the mechanistic insight into the wetting resistance-vapor permeability trade-off will pave the way for smart design of MD membranes in diverse water purification applications.

## Abstract (web summary)

Omniphobic membranes are attractive for membrane distillation because of their superior wetting resistance. Here the authors develop a particle-free approach for rapid fabrication of monolithic omniphobic membranes and elucidate the underlying mechanism of wetting resistance-vapor permeability trade-off in membrane distillation desalination.

## Introduction

Water scarcity is one of the most critical challenges of our time, posing a major threat to the global economy, regional stability, and ecosystem health^1–3^. The recent water crisis in the Southwest U.S.^4^ has caused enormous economic damage, and it is projected that 4–5 billion people will suffer from water stress globally by 2050^5^. To address this grand challenge, innovative technologies that enable the harvesting of purified water from unconventional water resources such as seawater, brackish water, and wastewater are indispensable^6,7^. Among others, membrane distillation (MD) has recently attracted great attention as an emerging desalination technology for water purification, due to its superior characteristics such as moderate operational temperature, high tolerance to salinity, and unique capability of utilizing low-grade thermal energy^8–10^. As a hybrid membrane-thermal process, MD utilizes the partial pressure gradient between hotter saline feedwater and colder permeate stream to drive the transport of water vapor across a microporous, hydrophobic membrane^11,12^. Maintaining membrane hydrophobicity is critical in MD, because it prevents salty feedwater from permeating through the membrane into the distilled water product (a phenomenon referred to as membrane wetting).

Conventional hydrophobic MD membranes (i.e., membranes that display apparent contact angle θ^*^ > 90° with high surface tension liquids such as water) suffer from membrane wetting in desalination of feedwater containing low surface energy contaminants (e.g., shale gas produced water^13,14^ and coal seam gas produced water^15,16^). Very recently, it has been demonstrated that membrane wetting induced by low surface energy contaminants can be significantly mitigated in MD by imparting omniphobicity to the membranes^8^. Unlike hydrophobic membranes, omniphobic membranes (i.e., membranes that display apparent contact angle θ^*^ > 90° with both high and low surface tension liquids) possess superior wetting resistance to liquids with a wide range of surface tensions. Omniphobic membranes are typically fabricated by combining reentrant texture and materials of low solid surface energy^17–22^. To date, the fabrication of omniphobic membranes involves complex and/or time-consuming processes, which typically require incorporation of micro- or nano-sized particles onto the membrane surface to create a hierarchical surface texture^13,16,23–29^. Also, the unintended environmental and health impacts of such particles, especially those with nano-scale sizes, continue to be an active area of research^30–32^. These concerns can potentially impede large-scale manufacturing of omniphobic membranes and consequently their applications in water purification. Further, water vapor permeability, a key parameter that characterizes the performance of membrane separation, has not received sufficient attention in the design of omniphobic MD membranes. A decrease in membrane water vapor permeability would increase both the cost and the energy consumption of desalination. However, little guidance exists on the relationship between membrane wetting resistance and water vapor permeability in the MD process. A fundamental understanding of such relationship, therefore, is of great significance to develop a design framework for smart MD membranes.

In this work, we present a particle-free approach that enables rapid fabrication (<1.5 h) of monolithic omniphobic polyvinylidene difluoride (PVDF) membranes for MD desalination. Our monolithic omniphobic membranes display excellent wetting resistance against liquids with low surface tensions (e.g., ethanol), as well as excellent water purification performance in direct contact MD of hypersaline solutions containing the surfactant sodium dodecyl sulfate (SDS). Further, we identify a trade-off between wetting resistance and water vapor permeability of our monolithic MD membranes and elucidate the underlying mechanisms. Analogous to the classic permeability-selectivity trade-off of synthetic membranes, which has directed the design criteria for membranes in desalination technologies including nanofiltration (NF), reverse osmosis (RO), and forward osmosis (FO)^33–35^, the trade-off we identified has the potential to profoundly impact the membrane design for MD process. We envision that our simple and rapid fabrication technique as well as our elucidation of the underlying mechanism of wetting resistance-vapor permeability trade-off will facilitate the practical use and smart design of omniphobic membranes in MD desalination and therefore contribute to the mitigation of water scarcity.

## Results

### Fabrication and characterization of omniphobic membrane

**Fig. 1:**
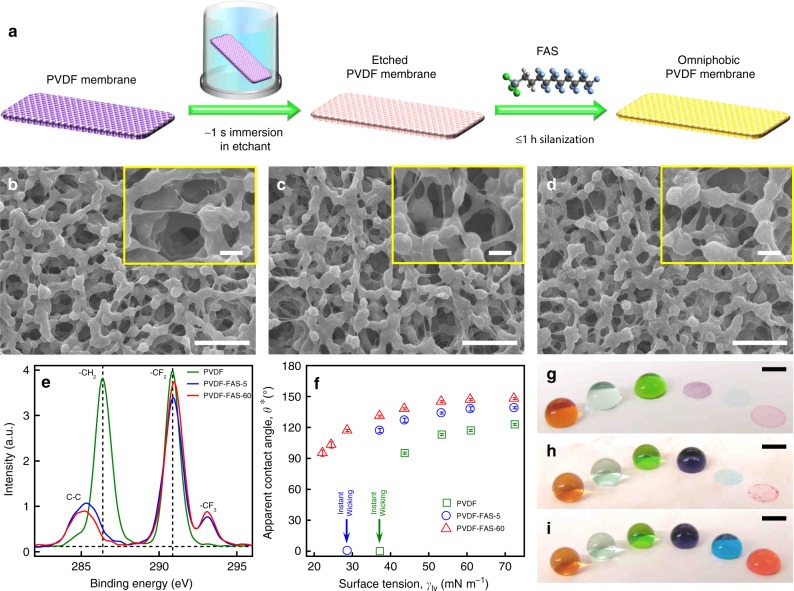
Fabrication and characterization of monolithic omniphobic membranes. **a** Schematic depicting the fabrication of omniphobic membranes. Scanning electron microscope (SEM) images of **b** pristine polyvinylidene difluoride (PVDF) membrane, **c** PVDF-FAS-5 membrane, and **d** PVDF-FAS-60 membrane. FAS refers to heptadecafluoro-1,1,2,2-tetrahydrodecyl trichlorosilane. The scale bars represent 5 and 1 μm (inset), respectively. **e** High resolution C1*s* XPS spectra of the membranes. **f** Apparent contact angles of liquids with different surface tensions on the membranes. Error bars represent standard deviation from three independent measurements. The green and blue arrows point to the surface tension at which liquid instantly wicked into pristine PVDF and PVDF-FAS-5 membranes, respectively. **g**–**i** Images showing different liquids beading up on or wetting **g** pristine PVDF membrane, **h** PVDF-FAS-5 membrane, and **i** PVDF-FAS-60 membrane. The droplets from left to right: water (*γ*_lv_ = 72.5 mN m^−1^), 1.5 mM sodium dodecyl sulfate (SDS) in water (*γ*_lv_ = 61 mN m^−1^), 20% ethanol in water (*γ*_lv_ = 43.7 mN m^−1^), 30% ethanol in water (*γ*_lv_ = 37.2 mN m^−1^), 60% ethanol in water (*γ*_lv_ = 28.7 mN m^−1^), 100% ethanol (*γ*_lv_ = 22.2 mN m^−1^). The scale bars represent 2 mm

PVDF membrane is one of the most commonly used membranes in MD process because of its inherent hydrophobicity, low thermal conductivity, and mechanical robustness^36^. However, hydrophobic PVDF membrane is prone to wetting, and surface engineering of PVDF membrane to improve its wetting resistance is a challenging task due to the chemical inertness of fluorocarbon materials. So far, complex and/or time-consuming processes^13,16,26,37^ have been used to activate PVDF membrane surface, followed by deposition of particles and surface fluorination, to render it omniphobic. In our approach to fabricate omniphobic PVDF membrane, ultra-fast etching of a commercial PVDF membrane (HVHP, Durapore) by immersing it in a sodium/naphthalene-based solution^38–40^ for ~1 s was combined with surface chemistry modification using a fluoroalkyl silane^41–44^ (heptadecafluoro-1,1,2,2-tetrahydrodecyl trichlorosilane, FAS) to impart low solid surface energy (see Fig. 1a; “Methods” section; Supplementary Movie 1). During the etching process, fluorine was stripped from the backbone of PVDF, while oxygen-containing functional groups such as hydroxyl and carboxyl groups were created to provide active sites for the subsequent grafting of fluoroalkyl silane via vapor-phase silanization^45,46^. This chemical transition was evident from the X-ray photon-electron spectroscopy (XPS) survey scans and high-resolution O1*s* spectra of pristine and etched PVDF membranes (see Supplementary Note 1; Supplementary Fig. 1). Different silanization durations (i.e., 5 min and 1 h) were applied to fabricate PVDF membranes with different wettability. The etched membranes after 5-min and 1-h silanization are designated as PVDF-FAS-5 and PVDF-FAS-60 membranes hereafter, respectively. The surface chemical compositions of these membranes were characterized with XPS and Fourier-transform infrared spectroscopy (FTIR) (see Fig. 1e; Supplementary Note 2; Supplementary Fig. 2). The high-resolution C1*s* XPS spectra (see Fig. 1e) indicated the presence of the characteristic –CH_2_ and –CF_2_ groups on the pristine PVDF membranes^47,48^. In contrast, the characteristic –CF_3_ group of fluoroalkyl silane was observed on the FAS-silanized PVDF membranes. In addition, PVDF-FAS-60 membrane possessed higher CF_3_/CF_2_ peak intensity ratio (0.236) than PVDF-FAS-5 membrane (0.227), indicating higher coverage of FAS on the surface and consequently lower solid surface energy.

Further, the pristine and processed membranes (see Fig. 1b–d) consisted of similar interconnected micro-sized PVDF granules with a reentrant texture and similar apparent surface pore size distributions obtained from SEM image analysis (see “Methods” section; Supplementary Note 3; Supplementary Fig. 3). The membrane pore size distributions were measured with a capillary flow porometer (see “Methods” section; Supplementary Note 4; Supplementary Fig. 4a–c), and similar membrane pore size distributions were also observed among the membranes. The mean membrane pore sizes were 0.452, 0.462, and 0.456 μm for the pristine PVDF, PVDF-FAS-5, and PVDF-FAS-60 membranes, respectively. These results indicate that the morphology of the processed PVDF membranes remains virtually unaltered compared to the pristine PVDF membrane. In addition, the air permeability of all the tested membranes was measured as an indicator of mass transfer resistance^49^. As shown in Supplementary Fig. 4d, the pristine and processed PVDF membranes displayed similar air permeability, indicating that the membrane modification employed in the current study did not result in additional mass transfer resistance.

The combination of the inherent reentrant texture of PVDF membrane with sufficient coverage of FAS possessing low solid surface energy rendered the PVDF-FAS-60 membrane omniphobic. Liquids with a wide range of surface tensions displayed high apparent θ^*^ on the omniphobic PVDF-FAS-60 membrane, including ethanol with an ultra-low surface tension (*γ*_lv_ = 22.2 mN m^−1^) demonstrating θ^*^ > 90° (see Fig. 1f and Supplementary Table 1). In contrast, the pristine PVDF membrane and PVDF-FAS-5 membrane were instantly wetted by water + 30% ethanol (*γ*_lv_ = 37.2 mN m^−1^) and water + 60% ethanol (*γ*_lv_ = 28.7 mN m^−1^), respectively. The different liquid repellency of the three PVDF membranes is evident from different arrays of liquids beading up on or wetting the membrane surfaces (see Fig. 1g–i). In addition, membrane filtration experiments were conducted to further demonstrate the distinct wetting resistance among the pristine and processed PVDF membranes. The membranes were sandwiched between two vertical glass tubes. A 12-cm column of water  + 30% ethanol (see Supplementary Movie 2) or 100% ethanol (see Supplementary Movie 3) was added to the upper tube. Both water + 30% ethanol and 100% ethanol permeated through the pristine PVDF membrane into the lower glass tube. While water + 30% ethanol could not permeate through the PVDF-FAS-5 membrane, 100% ethanol permeated through this membrane. In contrast, the PVDF-FAS-60 membrane displayed superior wetting resistance and neither water + 30% ethanol nor 100% ethanol could permeate through the membrane. Further, PVDF-FAS-60 membrane possesses higher liquid entry pressure (~175 ± 5 kPa) than that of PVDF-FAS-5 membrane (~146 ± 2 kPa) and pristine PVDF membrane (~114 ± 2 kPa) (see “Methods” section). Therefore, these results indicate that the order of wetting resistance was PVDF-FAS-60 membrane > PVDF-FAS-5 membrane > pristine PVDF membrane. It is worth noting that although PVDF-FAS-5 membrane was completely wetted by water + 60% ethanol, it was able to resist wetting of nonpolar liquids with even lower surface tensions, such as hexadecane (*γ*_lv_ = 27.5 mN m^−1^) and silicone oil (*γ*_lv_ = 21 mN m^−1^) (see Supplementary Table 1). This phenomenon highlights the importance of using polar liquids with low surface tensions to characterize membrane liquid repellency.

### Membrane wetting resistance in MD desalination

To evaluate desalination performance of the membranes with different surface wettability, we performed direct contact membrane distillation (DCMD) tests using hypersaline feed solution (1 M NaCl) supplemented with progressively increasing concentrations of SDS (see “Methods” section). The increase of SDS concentration lowered the surface tension of feed solutions, which would cause wetting of membranes with insufficient wetting resistance.

**Fig. 2:**
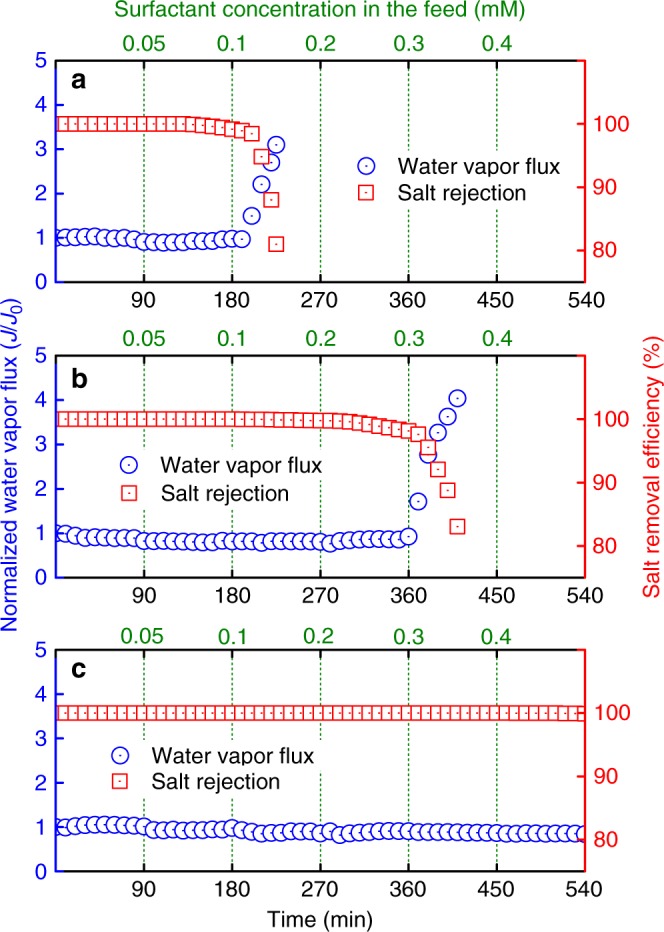
Membrane distillation (MD) performance of different PVDF membranes. Normalized water vapor flux (blue) and salt rejection (red) of **a** pristine PVDF membrane, **b** PVDF-FAS-5 membrane, and **c** PVDF-FAS-60 membrane in direct contact membrane distillation (DCMD) desalination, based on sequentially increasing doses of SDS. The feed solution contained 1 M NaCl, supplemented with various concentrations of SDS. The feed and distillate temperatures were maintained at 60 °C and 20 °C, respectively. Replicate results under identical experimental conditions are presented in Supplementary Fig. 5

All the membranes exhibited stable water vapor fluxes and perfect salt rejection prior to the addition of SDS (see Fig. 2; Supplementary Fig. 5), indicating successful desalination by allowing the transport of water vapor only. However, the water vapor flux of pristine PVDF membrane increased dramatically at 0.1 mM SDS (see Fig. 2a and Supplementary Fig. 5a), along with a substantial decrease of salt removal efficiency. This was because a large portion of the membrane pores was completely wetted by the feed solution, resulting in the penetration of dissolved salt into the distillate. The PVDF-FAS-5 membrane showed improved wetting resistance against 0.2 mM SDS, but still lost its desalination function at 0.3 mM SDS (see Fig. 2b and Supplementary Fig. 5b). In contrast, the omniphobic PVDF-FAS-60 membrane demonstrated remarkable wetting resistance and stable desalination performance even at 0.4 mM SDS (see Fig. 2c and Supplementary Fig. 5c). This was because the omniphobicity of the PVDF-FAS-60 membrane prevented complete penetration of saline feed solution with surfactants into the porous membrane structure. It should be noted that the highest SDS concentration resisted by our omniphobic membrane is comparable or higher than that reported in prior work with particle-incorporated, hierarchically structured omniphobic membranes^23,24,27–29^, indicating that a monolithic membrane with reentrant texture is sufficient to achieve omniphobicity in MD desalination.

### Wetting resistance and water vapor permeability trade-off

**Fig. 3:**
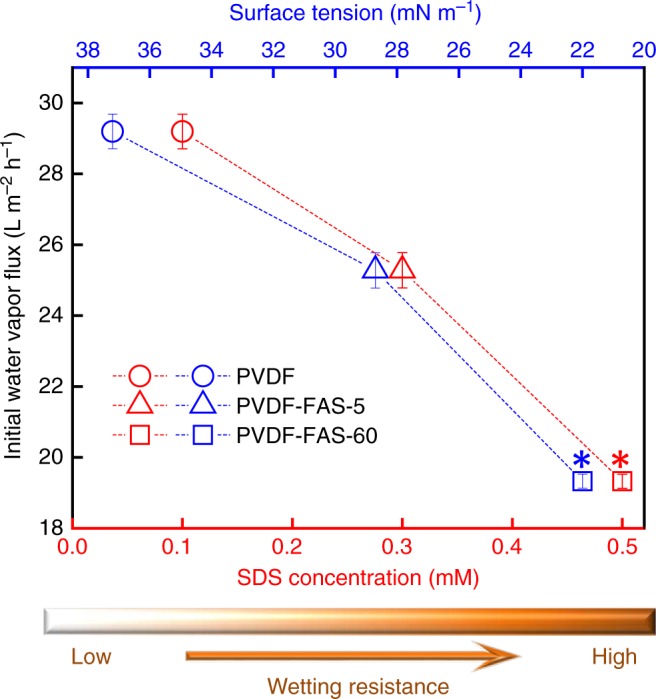
The relationship between initial water vapor flux and wetting resistance of different MD membranes tested in this study. The blue and red symbols refer to the critical surface tension and the critical SDS concentration, respectively, at which each membrane was completely wetted. Membranes with enhanced wetting resistance tolerate liquids with lower surface tensions and higher concentrations of SDS. The asterisks indicate that our omniphobic membrane was indeed not fully wetted by ethanol (in contact angle measurement) or 0.4 mM SDS (in DCMD desalination). Error bars represent standard deviation from three independent measurements

Ideally, membranes possessing both robust wetting resistance and high water vapor permeability are desirable in the MD process. However, our pristine PVDF, PVDF-FAS-5, and PVDF-FAS-60 membranes displayed decreasing water vapor permeability with increasing wetting resistance (see Fig. 3). A thorough literature search indicates a similar phenomenon—omniphobic MD membranes with higher wetting resistance typically possess lower water vapor permeability compared to hydrophobic MD membranes with lower wetting resistance (see Supplementary Table 2)^13,16,24–26,28,29,50,51^. This phenomenon is intriguing because wetting resistance (an inverse measure of ease of liquid permeation) and water vapor permeability (a measure of ease of water vapor permeation) are distinct properties. The mechanism of this phenomenon is rarely addressed in the literature. While a few studies qualitatively attributed it to altered membrane morphology (e.g., increased membrane thickness and decreased pore sizes)^16,26^, such arguments cannot explain our results because no morphological difference was observed for our membranes (see Fig. 1b–d), regardless of their wetting resistance and water vapor permeability. In addition, the results of air permeability measured at a wide range of pressures (see Supplementary Fig. 4d) indicate that our PVDF-FAS-5 and PVDF-FAS-60 membranes with higher wetting resistance do not possess additional mass transfer resistance compared to the pristine PVDF membrane. So, there is a need to understand the relationship between wetting resistance and water vapor permeability of MD membranes mechanistically.

In order to elucidate the mechanisms underlying the wetting resistance-water vapor permeability trade-off of our monolithic membranes, let us first consider the breakthrough pressure *P*_b_^52^ (i.e., the pressure at which liquid transitions from the nonwetting Cassie–Baxter state^53^ to the wetted Wenzel state^54^) of individual pores with different sizes for PVDF membranes. Assuming that the membranes are composed of hexagonally arranged spherical features with diameters 2*R* and pore sizes (i.e., inter-feature spacing) 2*D* (see Supplementary Note 6), the breakthrough pressure *P*_b_ of each pore can be determined from a force balance at the liquid–air interface:^55^

1 $$P_{\mathrm{b}} \approx \frac{{4\pi \gamma _{{\mathrm{lv}}}(1 - {\mathrm{cos}}\theta )}}{{R(2\sqrt 3 D^ \ast - \pi )(\sqrt {D^ \ast } - 1 + 2{\mathrm{sin}}\theta )}}$$

**Fig. 4:**
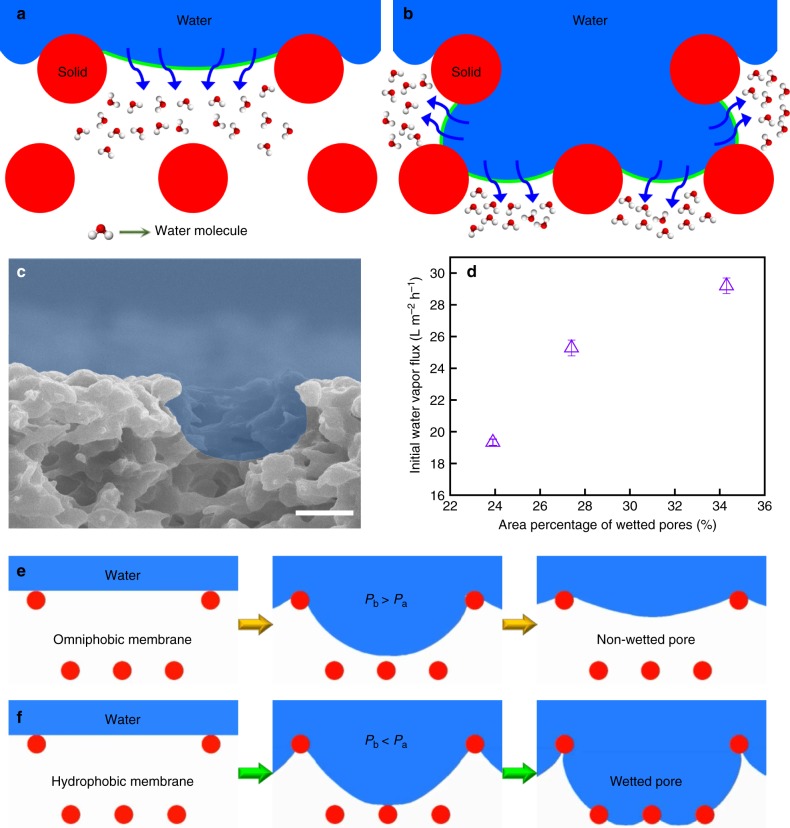
Mechanism underlying wetting resistance-water vapor permeability trade-off in MD desalination with monolithic omniphobic membranes. **a**, **b** Schematics (not drawn to scale) showing that the effective water–air interfacial area for evaporation (solid green line) increases when feedwater wets the pores. **c** Cross-sectional view of PVDF membrane surface, with the pore larger than the critical pore size for wetting (i.e., wetted pore depicted in blue). The scale bar represents 2 μm. **d** The positive correlation observed between area fraction of wetted pores and the initial water vapor flux of the membranes. Error bars represent standard deviation from three independent measurements. **e**, **f** A series of snapshots from numerical simulations, showing dynamic formation of **e** nonwetted and **f** wetted pores on omniphobic membrane (*P*_b_ ≈ 1.8 kPa > *P*_a_ ≈ 1.2 kPa for the first layer) and hydrophobic membrane (*P*_b_ ≈ 1.19 kPa < *P*_a_ ≈ 1.2 kPa for the first layer), respectively. Water cannot permeate through the second layer because *P*_b_ (~29.4 and ~19.5 kPa for the second layer of omniphobic and hydrophobic membranes, respectively) was greater than *P*_a_. Note that *P*_a_ refers to applied pressure. *P*_b_ refers to breakthrough pressure, which can be estimated using Eq. (1)

Here, *θ* is the Young’s contact angle, and the dimensionless parameter, D* = [(*R* + *D*)/*R*]^2^, is a measure of the air trapped underneath a liquid droplet when it forms a composite interface with a textured surface. It is evident from Eq. (1) that the breakthrough pressure decreases with increasing the pore sizes of the membrane. When the membrane pore size exceeds a certain threshold, the corresponding *P*_b_ becomes lower than the transmembrane pressure, leading to the wetting of these pores due to the permeation of liquid water. Therefore, for a membrane with nonuniform pore size distribution (such as the PVDF membranes considered in this work), larger pores with breakthrough pressure less than transmembrane pressure become wetted in the MD process, while smaller pores with breakthrough pressure larger than transmembrane pressure remain nonwetted. More importantly, for a given pore size, the breakthrough pressure decreases with increasing the wettability (i.e., decreasing Young’s contact angle). Consequently, for membranes with the same pore size distribution but different wettability, the hydrophobic membrane with lower wetting resistance (e.g., the pristine PVDF membrane) possesses more wetted pores compared to the omniphobic membrane with higher wetting resistance (e.g., the PVDF-FAS-60 membrane) in MD desalination. Compared to nonwetted pores with smaller water–air interfacial area (leading to one-dimensional evaporation, see Fig. 4a), the water-filled wetted pores provide larger water–air interfacial area (leading to more effective three-dimensional evaporation, see Fig. 4b). Therefore, the hydrophobic membrane with more wetted pores is expected to display higher water vapor flux than the omniphobic membrane.

Based on Eq. (1), for the transmembrane pressure of 1.2 kPa in our DCMD system, we estimated the critical pore sizes for wetting (beyond which the pores become wetted due to permeation of liquid water; see Fig. 4c) of pristine PVDF, PVDF-FAS-5, and PVDF-FAS-60 membranes to be 3.35, 3.65, and 3.88 μm, respectively. Correspondingly, we estimated the wetted pore area fractions (obtained from the apparent surface pore size distribution in Fig. S3; see “Methods” section; Supplementary Note 3) of pristine PVDF, PVDF-FAS-5, and PVDF-FAS-60 membranes to be ~34.3%, 27.4%, and 23.9%, respectively (see Fig. 4d). Lower wetted pore area fraction implies smaller water–air interfacial area for evaporation, which in turn results in lower water vapor flux. Consequently, for pristine PVDF, PVDF-FAS-5, and PVDF-FAS-60 membranes, while the wetting resistance increased (see Fig. 1f), the water vapor flux decreased (see Fig. 4d).

To further elucidate the dynamic formation of nonwetted and wetted pores on membranes with different wetting resistance, we performed numerical simulations to reveal the evolution of water–air interface upon water contacting membrane surface under an applied pressure (see “Methods” section and Supplementary Movies 4 and 5). A two-layer porous structure consisting of spherical features was used to represent the membrane structure (see Fig. 4e, f). When an applied pressure *P*_a_ (i.e., transmembrane pressure of 1.2 kPa) was exerted on water at rest on the first layer, the water–air interface deformed and moved toward the second layer. When *P*_b_ of the membrane pore was greater than *P*_a_ (e.g., PVDF-FAS-60 omniphobic membrane), our numerical simulations indicate that water cannot completely permeate through the first layer of the membrane, and a stable water–air interface is eventually formed (see Fig. 4e). This leads to the formation of a nonwetted pore on the omniphobic membrane. In contrast, when *P*_b_ of the membrane pore, with same geometry, was less than *P*_a_ (e.g., PVDF hydrophobic membrane), our numerical simulations indicate that water permeates through the first layer of the membrane, and forms wetted pores with larger water–air interfacial area (see Fig. 4f). These numerical simulation results are consistent with our schematic explanation depicted in Fig. 4a, b, which indicate that membrane wettability regulates water vapor permeability through the effective evaporation area. In other words, hydrophobic membrane with more wetted pores (see Supplementary Movie 6) is expected to display higher water vapor flux than the omniphobic membrane with less wetted pores (see Supplementary Movie 7).

## Discussion

In this work, we fabricated monolithic omniphobic membranes, which displayed both excellent repellency to low surface tension liquids (including ethanol) and robust wetting resistance against surfactants in MD desalination. Compared to the fabrication of particle-incorporated omniphobic membranes, which typically require multiple steps and lengthy preparation duration (e.g., from hours to days), our facile and particle-free approach enables rapid (<1.5 h) and scalable processing of omniphobic membranes. The monolithic feature of our membranes avoids potential detachment of particles, and thus improves membrane reliability in the MD process. Therefore, our fabrication approach has great potential to achieve large-scale manufacturing of omniphobic membranes for MD desalination.

More importantly, a trade-off between wetting resistance and water vapor permeability of MD membranes was identified in our study (see Fig. 3; and Supplementary Note 7; Supplementary Fig. 7). Such a trade-off has important implications that influence the membrane design and selection for MD desalination. Although omniphobic membranes demonstrate superior wetting resistance in MD process, this performance gain is offset by their reduced water vapor permeability that hinders process efficiency. On the other hand, MD membranes with high water vapor permeability tend to have inferior wetting resistance, rendering those membranes inappropriate for the treatment of wastewater with low surface tension. This dilemma is analogous to the classic permeability-selectivity trade-off in membrane desalination, in which an increase of water permeability typically leads to lower membrane selectivity (i.e., reduced salt removal efficiency^33–35^). Both trade-offs suggest that the design of appropriate membranes for desalination requires balance and optimization among different membrane properties. Current research efforts are investing heavily in fabrication of novel omniphobic membranes for MD^8^. However, achieving membrane omniphobicity at the expense of water vapor permeability might not be beneficial in MD desalination, particularly when relatively low concentrations of low surface energy contaminants are present in the feedwater. The practical impacts of membrane wettability on MD performance should, therefore, be reevaluated by taking membrane water production into consideration. In other words, one needs to consider membrane wetting resistance and water vapor permeability comprehensively^15,49,56,57^ in designing membrane materials for MD desalination of different feedwaters.

In summary, we developed a simple, scalable, and particle-free approach that enables rapid processing (<1.5 h) of monolithic omniphobic PVDF membranes, and demonstrated a wetting resistance-vapor permeability trade-off for our monolithic membranes in MD desalination. We believe that our fabrication method has promising potential to simplify the manufacturing and scale-up of omniphobic MD membranes. Further, we envision that the wetting resistance-permeability trade-off as well as the mechanistic insight conveyed in our work will pave the way for smarter design strategies for high-performance MD membranes, thereby promoting the cost- and energy-efficiencies of MD desalination for water purification.

## Methods

### Fabrication of monolithic omniphobic membranes

A sodium/naphthalene-based etching solution with 2-methoxyethyl ether as the solvent (FluoroEtch, Acton Technologies) was used to etch flat sheet polyvinylidene fluoride (PVDF) membranes with a nominal pore size of 0.45 μm (HVHP, Durapore). The PVDF membranes were immersed in the etching solution for ~1 s (see Supplementary Movie 1). It should be noted that the membranes were completely wetted by the etching solution upon immersion. Immediately after the membranes were taken out of the etching solution, the etched membranes were thoroughly washed with isopropanol, 0.1 mM acetic acid aqueous solution (~65 °C), and deionized water in sequence. The entire process of immersing and washing took ~1.5 min. The membranes were dried using nitrogen gas and by heating at 80 °C for 20 min. Subsequently, the processed PVDF membranes were modified via vapor-phase silanization at 90 °C using heptadecafluoro-1,1,2,2-tetrahydrodecyl trichlorosilane (FAS, Gelest) to impart low solid surface energy. The membranes were then thoroughly rinsed with n-hexane. Different durations of silanization (i.e., 5 and 60 min) were employed to impart different degrees of wetting resistance to the PVDF membrane.

### Characterization of membrane surface morphology

The surface morphology of the pristine and processed PVDF membranes was characterized using a scanning electron microscope (SEM; JEOL JSM-6500F) at 10 kV. The surface pore sizes were analyzed with ImageJ (National Institutes of Health). The grayscale SEM image was first converted to a binary (i.e., black and white) image (see Supplementary Note 3). The apparent surface pores with irregular shapes were then automatically identified with ImageJ. For each apparent surface pore, the Feret’s diameter (i.e., the longest distance between any two points on the boundary of the surface pore) was measured as the apparent surface pore size. We used Feret’s diameter to characterize the apparent surface pore because the permeation of liquid into a pore with irregular shape depends on the largest dimension of the surface pore^52,58–60^. For each membrane, ~2000 individual pores obtained from three different SEM images were analyzed to obtain the apparent surface pore size distribution.

### Characterization of membrane with capillary flow porometry

The pristine and processed PVDF membranes were characterized with a capillary flow porometer (Model CFP-1100A) at Porous Materials Inc. to measure the membrane pore size and the air permeability (see Supplementary Note 4). Wet/dry flow method was used to measure the membrane pore diameter and dry flow method was used to measure the permeability of air at different pressures. Galwick fluid with a surface tension of 15.9 mN m^−1^ was used as the wetting liquid to completely wet all tested membranes (i.e., the contact angles of Galwick on all our membranes were 0°).

### Measurement of liquid entry pressure

The liquid entry pressure of each membrane was measured by placing the membrane in a dead end filtration cell (UHP-43, Sterlitech);^15,49^ the cell was then filled with 50 ml DI water and tightly sealed. Subsequently, the cell was pressurized with compressed air in a step-wise manner (increment of 5 ± 1 kPa and ~5 min for stabilization after each increment). The pressure at which the first water droplet completely permeated through the membrane and flowed out of the cell was measured as the liquid entry pressure. Three independent measurements were conducted for each membrane.

### Characterization of surface chemical composition

XPS analysis was performed on the membrane surface using a PHI-5800 spectrometer (Physical Electronics) with a monochromatic Al-K X-ray source operated at 15 kV. The photoelectrons were collected at a takeoff angle of 45° relative to the membrane surface. FTIR spectroscopy was performed with a Nicolet iS-50 spectrometer (Thermo Fisher Scientific).

### Measurement of contact angles

The apparent contact angles of liquids with a wide range of surface tensions (21–72.5 mN m^−1^) were measured using a contact angle goniometer (Ramé-Hart 200-F1). By mixing DI water (72.5 mN m^−1^) with different concentrations of pure ethanol (22.2 mN m^−1^), we were able to create an array of polar liquids with gradually decreasing surface tension (see Supplementary Table 1). For each liquid, three independent measurements with ~8 μL droplets were performed on each membrane.

### Membrane distillation of feed solutions with surfactants

The membrane wetting resistance against SDS was evaluated with a custom-built DCMD system with a transparent acrylic cell. SDS is a representative substance that has been typically used to assess membrane wetting resistance in the MD process in the literature^16,27,28^. The feedwater and distillate channels of the acrylic cell had an identical dimension of 77 mm × 26 mm × 3 mm, corresponding to an effective membrane area of 20.02 cm^2^. The temperatures of the feed solution and deionized distillate were kept at 60 °C and 20 °C, respectively, using two recirculating water baths (Polystat, Cole-Parmer). The crossflow velocities of the feed and distillate streams were 9.6 cm s^−1^ (0.45 L min^−1^) and 5.3 cm s^−1^ (0.25 L min^−1^) in a concurrent mode, respectively. During the initial 90 min of MD desalination, 1 M NaCl solution at 60 °C was used as the feedwater. SDS was then introduced to the feed reservoir every 90 min to progressively increase the SDS concentration and consequently lower the surface tension of the feed solution. The SDS concentrations after sequential additions were 0.05, 0.1, 0.2, 0.3, and 0.4 mM. Water vapor flux across the membrane (*J*_w_) was measured by monitoring the weight of the solution in the distillate reservoir using a digital balance (EW-10001-05, Cole-Parmer). The salt rejection efficiency was calculated from NaCl concentration in the permeate measured by a calibrated conductivity meter (Oakton Instruments).

### Numerical simulations

Two-dimensional numerical simulations with an incompressible, laminar flow model were conducted to reveal the evolution of water–air interface upon water contacting the membrane under an applied pressure (i.e., the transmembrane pressure of 1.2 kPa in the MD experiment, as measured by a low-pressure gauge). The membrane was modeled as a two-layer porous structure consisting of spherical features. The inter-feature spacing of the spherical features was set to represent the critical pore size of the pristine PVDF membrane. The contact angle hysteresis was ignored on the membrane surface in the simulation. We solved the governing equations with computational fluid dynamics software ANSYS Fluent using a pressure-based solver. The geometric reconstruction scheme was used in the volume of fluid model to represent the liquid–air interface. The continuum surface force method was employed in the momentum equation. The SIMPLE (semi-implicit, explicit) algorithm was used for pressure-velocity coupling. A variable time step scheme was used to ensure Courant–Friedrich–Levy number <1.0 in each time step. Iterations at each time step were terminated when the convergence criteria of all equations were smaller than 10^−6^.

## Supplementary information

Supplementary Information

Description of Additional Supplementary Files

Supplementary Movie 1

Supplementary Movie 2

Supplementary Movie 3

Supplementary Movie 4

Supplementary Movie 5

Supplementary Movie 6

Supplementary Movie 7

## Data Availability

The authors declare that the data supporting the findings of this study are available within the article and its Supplementary Information files and also are available from the corresponding authors upon reasonable request.
