# Supplementary material for: Trade-off in membrane distillation with monolithic omniphobic membranes
Source: Nat Commun. 2019 Jul 19;10:3220. doi: 10.1038/s41467-019-11209-6 (PMC6642111; doi:10.1038/s41467-019-11209-6)
Supplement: Supplementary file 1 — Supplementary Information [file 41467_2019_11209_MOESM1_ESM.pdf]

Supplementary Information

Trade-off in Membrane Distillation with Monolithic Omniphobic Membranes

Wang et al.

Supplementary Note 1. Characterization of surface chemical composition of pristine and etched PVDF membranes with XPS

In order to characterize the surface chemical composition of the pristine PVDF membrane and the etched PVDF membrane, we obtained the X-ray photon-electron spectroscopy (XPS) survey scans and high-resolution O1*s* spectra (see Supplementary Figure 1). The etched PVDF membrane had a higher peak intensity ratio of C/F (~0.5) than pristine PVDF membrane (~0.18) based on the XPS survey scans. Compared to the pristine PVDF membrane, the etched PVDF membrane also possessed abundant oxygen-containing functional groups.

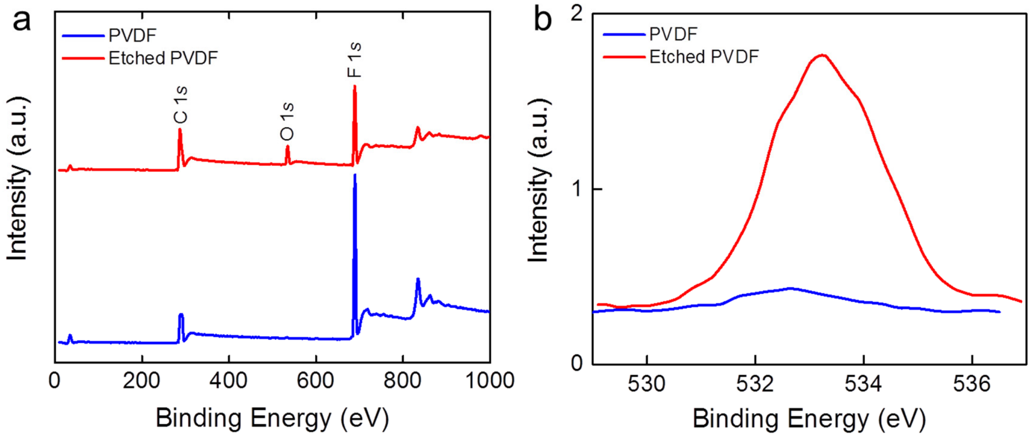

Supplementary Figure 1. X-ray photon-electron spectroscopy (XPS) characterization of the pristine and etched polyvinylidene difluoride (PVDF) membranes. (a) XPS survey scans and (b) high-solution O1*s* XPS spectra of pristine PVDF membrane and etched PVDF membrane.

Supplementary Note 2. Characterization of chemical composition of PVDF-FAS-5 and PVDF-FAS-60 membranes with XPS and FTIR

In order to characterize the surface chemical composition of PVDF-FAS-5 and PVDF-FAS-60 membranes, we obtained the XPS survey scans (see Supplementary Figure 2a). Compared to pristine PVDF membrane that possesses only carbon and fluorine on the surface, the PVDF-FAS-5 and PVDF-FAS-60 membranes possess additional silicon and oxygen peaks due to the grafting of fluoroalkyl silane onto the membrane surface.

Further, Fourier-transform infrared spectroscopy (FTIR) was performed to characterize the functional groups of the membranes. The spectra of PVDF membranes were consistent with the literature (see Supplementary Figure 2b).[1] For example, the absorbance peaks at approximately 833 cm^{-1}, 876 cm^{-1}, 1072 cm^{-1}, 1171/1232 cm^{-1}, and 1402 cm^{-1} correspond to crystal phase vibration, C-C skeleton vibration, crystal phase vibration, -CF_2 stretching vibration, and -CH_2 vibration, respectively.[1,2] As might be anticipated, no significant difference was observed among the FTIR spectra of the membranes due to the deeper penetration depth in FTIR spectroscopy.[3]

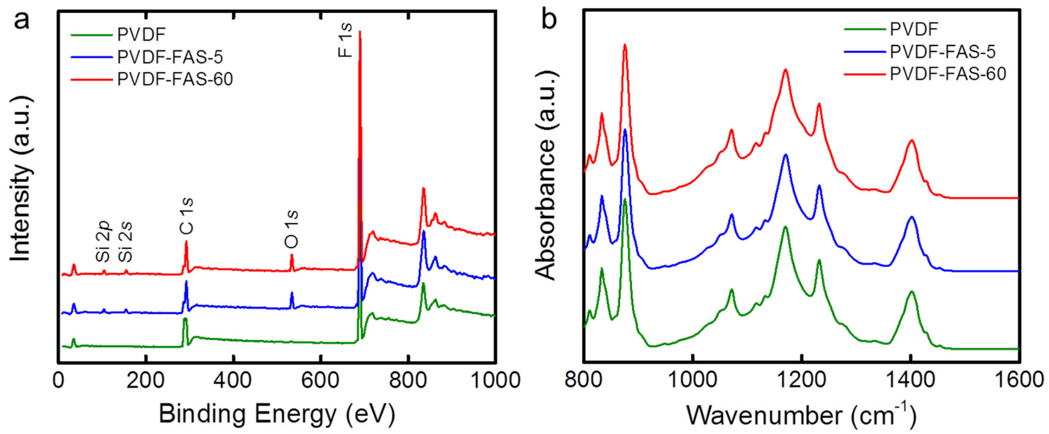

Supplementary Figure 2. Membrane surface characterization. (a) XPS survey scans and (b) Fourier-transform infrared spectroscopy (FTIR) spectra of pristine PVDF membrane, PVDF-FAS-5 and PVDF-FAS-60 membranes. FAS refers to heptadecafluoro-1,1,2,2-tetrahydrodecyl trichlorosilane.

Supplementary Note 3. Apparent surface pore size distributions of pristine and etched PVDF membranes

The surface morphology of the pristine and the processed PVDF membranes was characterized using a scanning electron microscope. The pristine and processed membranes consisted of interconnected micro-sized PVDF granules with a re-entrant texture (see Supplementary Figure 3a-3c). The apparent surface pore sizes as well as the feature sizes of the membranes were measured by analyzing the SEM images with ImageJ (National Institutes of Health). The grayscale SEM image (see Supplementary Figure 3d) was first converted to a binary (i.e., black and white) image (see Supplementary Figure 3e). The surface pores with irregular shapes were then automatically identified using ImageJ (see Supplementary Figure 3f). For each surface pore, the Feret's diameter (i.e., the longest distance between any two points on the boundary of the surface pore) was measured as the apparent surface pore size. We used Feret's diameter to characterize the surface pore because the permeation of liquid into a pore with irregular shape depends on the largest dimension of the surface pore.[4-7] For each membrane, approximately 2000 individual pores obtained from 3 different SEM images were analyzed to obtain the apparent surface pore size distribution. The similar apparent surface pore size distributions (see Supplementary Figure 3g-3i) indicate that the morphology of the PVDF membranes remained unaltered after the etching and silanization processes.

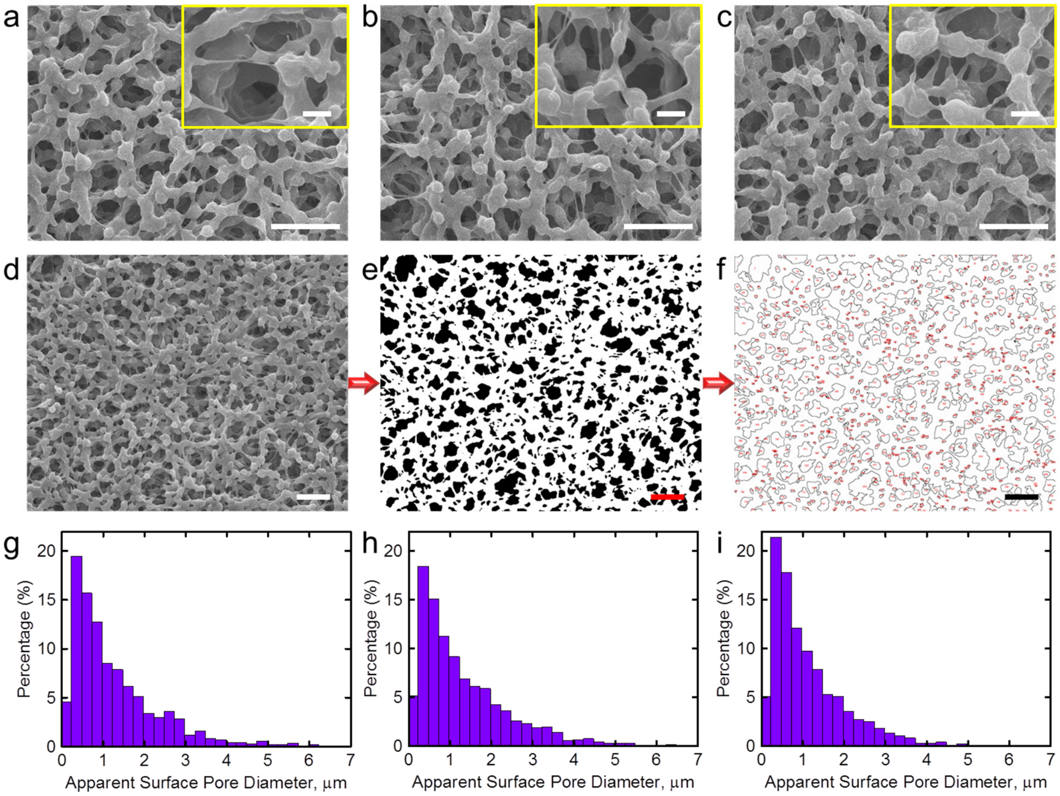

Supplementary Figure 3. Scanning electron microscope (SEM) analysis of PVDF membranes. (a-c) SEM images of (a) pristine PVDF membrane, (b) PVDF-FAS-5 membrane, and (c) PVDF-FAS-60 membrane. The scale bars represent 5 μm and 1 μm (inset), respectively. (d-f) Grayscale SEM image (d) was converted to a binary image (e). The apparent surface pores (f) were then identified with ImageJ. The scale bars represent 5 μm. (g-h) Apparent surface pore size distribution of (g) pristine PVDF membrane, (h) PVDF-FAS-5 membrane, and (i) PVDF-FAS-60 membrane.

Supplementary Note 4. Characterization of pristine and processed PVDF membranes with capillary flow porometry

The membranes were characterized with a capillary flow porometer (Model CFP-1100A) at Porous Materials Inc. to measure the membrane pore size and air permeability. The membrane mean pore sizes, measured with the wet/dry flow method, are 0.452 μm, 0.462 μm, and 0.456 μm for the pristine PVDF, PVDF-FAS-5, and PVDF-FAS-60 membranes, respectively. Also, all the tested membranes display similar membrane pore size distributions (see Supplementary Figure 4a-4c). These results indicate that the morphology of the processed PVDF membranes remains virtually unaltered compared to the pristine PVDF membrane. It is worth noting that the membrane pore size distributions measured with capillary flow porometry are narrower than the apparent surface pore size distributions (see Supplementary Figure 3g-3i) obtained from SEM image analysis. This is because the membrane pore size measured with capillary flow porometry represents the throat diameter (i.e., the most constricted/the smallest distance), along the pore.[8-10] Additionally, the air permeability measured at a wide range of pressures (see Supplementary Figure 4d) indicates that our processed membranes do not display additional mass transfer resistance compared to the pristine PVDF membrane.

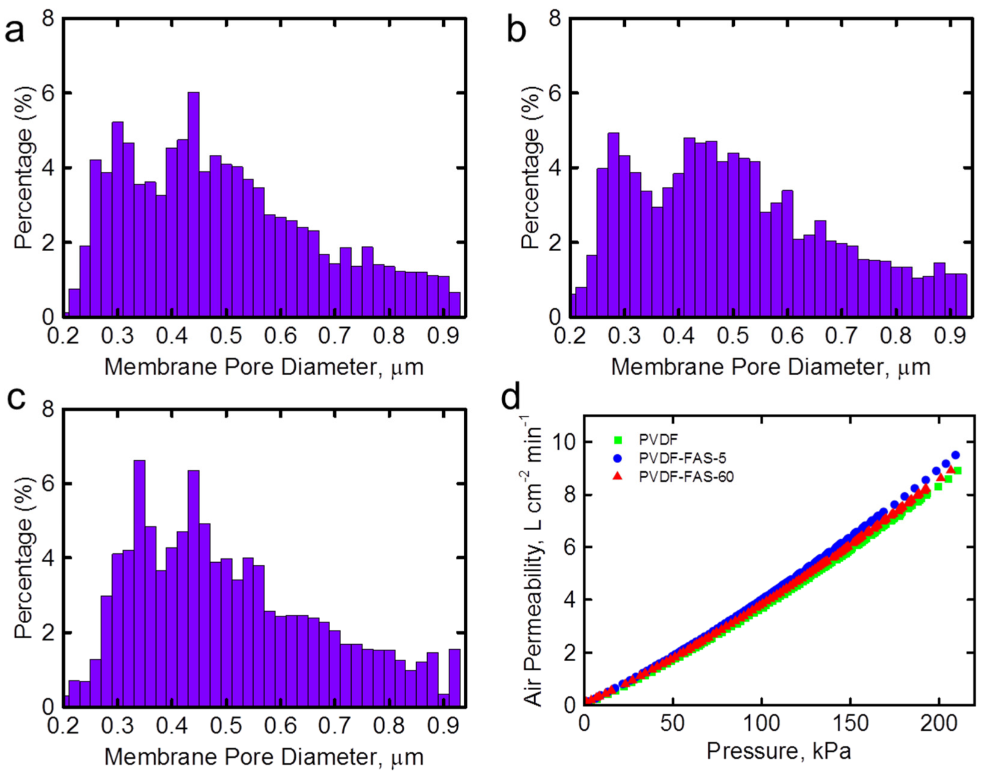

Supplementary Figure 4. Membrane characterization with capillary flow porometry. (a-c) Membrane pore size distribution of (a) pristine PVDF membrane, (b) PVDF-FAS-5 membrane, and (c) PVDF-FAS-60 membrane. (d) Air permeability of the tested membranes measured at different pressures.

Supplementary Note 5. Contact angles of different liquids

The apparent contact angles of ~8 μL droplets of liquids with different surface tensions on the pristine PVDF membrane and processed PVDF membranes with 5 min silanization (PVDF-FAS-5) and 1 h silanization (PVDF-FAS-60) are summarized in Supplementary Table 1. It is worth noting that the PVDF-FAS-5 membrane was completely wetted by water+60% ethanol (γ_{lv} = 28.7 mN m^{-1}), water+80% ethanol (γ_{lv} = 24.5 mN m^{-1}) and 100% ethanol (γ_{lv} = 22.2 mN m^{-1}), which were polar liquids. However, it displayed good wetting resistance against non-polar liquids with lower surface tensions, such as hexadecane (γ_{lv} = 27.5 mN m^{-1}) and silicone oil (γ_{lv} = 21 mN m^{-1}). These results highlight the importance of using polar liquids with low surface tension to accurately assess the wetting resistance of MD membranes.

In addition to the three membranes discussed above, we also measured the apparent contact angles of liquids on another membrane designated as PVDF-FAS-E (E refers to extended immersion time in the etchant solution). This membrane was fabricated in the same way as PVDF-FAS-60 membrane, except that the immersion time in the etchant solution was extended to ~30 s. Our results indicate that the apparent contact angles of liquids on PVDF-FAS-60 membrane and PVDF-FAS-E membrane (see Supplementary Table 1) are similar, indicating that the extended immersion time (~30 s) in the etchant solution did not alter the omniphobicity of the membrane.

Supplementary Table 1. The apparent static contact angles θ^*, apparent advancing θ^*_{adv} and apparent receding θ^*_{rec} contact angles of various liquids on the pristine PVDF, PVDF-FAS-5, PVDF-FAS-60, and PVDF-FAS-E membranes. The errors in contact angle measurements were ≤ 3°.

		Pristine PVDF			PVDF-FAS-5			PVDF-FAS-60			PVDF-FAS-E		
Liquid	γ_{lv} (mN m^{-1})	θ^*	θ^*_{adv}	θ^*_{rec}	θ^*	θ^*_{adv}	θ^*_{rec}	θ^*	θ^*_{adv}	θ^*_{rec}	θ^*	θ^*_{adv}	θ^*_{rec}
Water	72.5	123°	138°	20°	139°	155°	45°	149°	160°	88°	150°	160°	87°
1.5 mM SDS	61	117°	130°	13°	138°	152°	14°	147°	155°	82°	147°	154°	80°
10% ethanol	53.4	113°	119°	8°	134°	148°	11°	145°	153°	43°	146°	153°	45°
20% ethanol	43.7	95°	99°	0°	127°	133°	9°	138°	149°	15°	138°	150°	13°
30% ethanol	37.2	0°	0°	0°	117°	122°	0°	131°	142°	10°	130°	140°	8°
60% ethanol	28.7	0°	0°	0°	0°	0°	0°	117°	128°	0°	119°	127°	0°
80% ethanol	24.5	0°	0°	0°	0°	0°	0°	103°	115°	0°	104°	115°	0°
100% ethanol	22.2	0°	0°	0°	0°	0°	0°	95°	100°	0°	93°	97°	0°
Hexadecane	27.5	0°	0°	0°	112°	118°	0°	117°	132°	0°	116°	129°	0°
Silicone oil	21	0°	0°	0°	99°	103°	0°	109°	115°	0°	109°	116°	0°

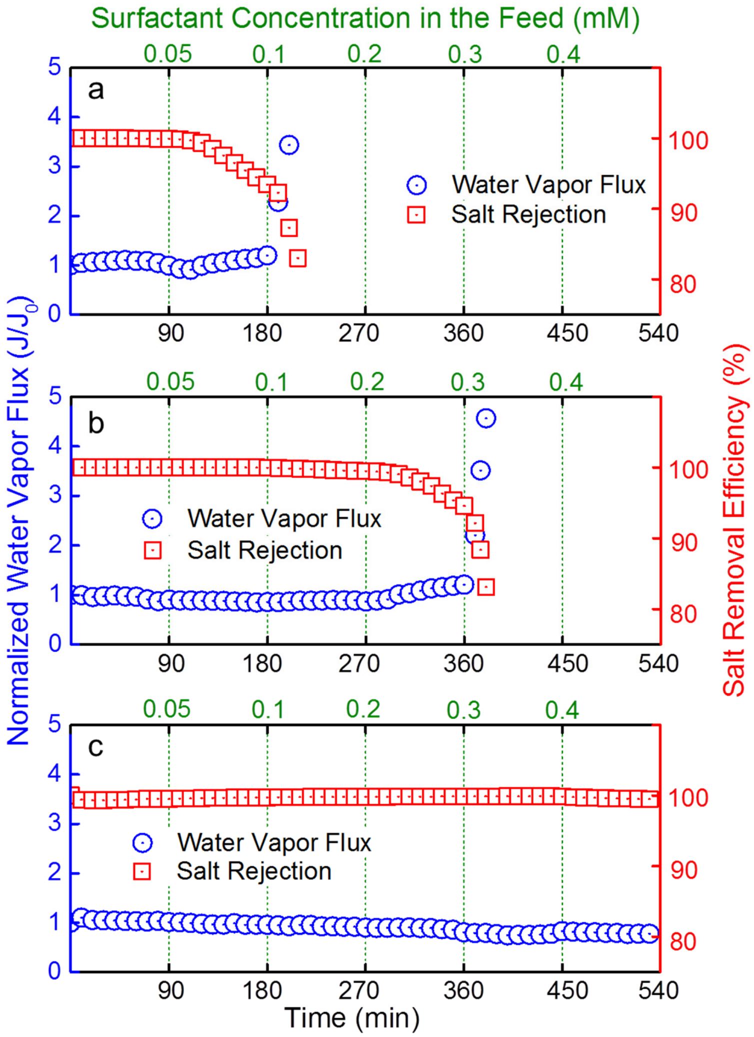

Supplementary Figure 5. Membrane distillation (MD) performance of different PVDF membranes. Normalized water vapor flux (blue) and salt removal efficiency (red) of (a) pristine PVDF membrane, (b) PVDF-FAS-5 membrane, and (c) PVDF-FAS-60 membrane in direct contact membrane distillation (DCMD) desalination, based on sequential increasing doses of sodium dodecyl sulfate (SDS). The feed solution contained 1 M NaCl, supplemented with various concentrations of SDS. The feed and distillate temperatures were maintained at 60°C and 20°C, respectively. This figure serves as a replicate of the results presented in Fig. 3 of the main text.

Supplementary Table 2. Comparison of water vapor permeability between omniphobic and hydrophobic membranes in MD desalination process. While the observed trade-off in prior studies could be attributed to altered membrane morphology (e.g., increased membrane thickness and decrease pore sizes), this cannot explain the trade-off observed in our monolithic membranes and indicates the importance of considering water vapor permeability in the membrane design for MD process.

Pristine Membrane		Omniphobic Membrane		Membrane Distillation Tests			Reference
Material	Water Contact Angle (°)	Fabricated Procedure	Water Contact Angle (°)	Feed Solution	Temp. (F/P [a], °C)	Initial Water Flux (F/P, L m^{-2} h^{-1})	
PVDF-HEP	136.4 ± 2.4	Grafting with fluorinated SiNPs [b]	156.9 ± 0.8	35 g/L NaCl	60/20	34.1/13.3	Huang[11]
PVDF-HEP	~130	Grafting with fluorinated SiNPs	~150	1 M NaCl	60/20	25/12.5	Lee[12]
PVDF	68.9 ± 2.3	Grafting with fluorinatedsilica aerogel via LBL [c] deposition	177.0 ± 0.4	RO brine from coal seam gas produced water	60/20	13.17/11.22	Woo[13]
PVDF	126.7± 1.4	Grafting with fluoribated hierarchical SiNPs@PS [d] spheres	176.5°± 0.1	An emulsion composed of SDS: hexadecane: NaCl = 240:2400:10000 (mg/L) in water	60/20	11/9	Zheng[14]
PVDF	~110	Grafting with fluorinated SiNPs	>150	1 M NaCl	60/20	23.5/13.6	Boo[1]
PVDF	124.2	Deposition of with fluorinated SiNPs	167.3	3.5 wt% NaCl solution	76/21	30/21	Lu-2017[15]
PVDF-HEP	132.1 ± 0.9	Electrospinning of PVDF-HEP and (F-POSS [e]) colloidal suspension solution.	154.5 ± 2.6	1 M NaCl	60/20	11/9	Lu-2018a[16]
PVDF	~117	Grafting with fluorinated SiNPs	~145	3.5 wt% NaCl solution	70/Liquid nitrogen (vacuum membrane	24.9/14.6	Lu-2018b[17]

					distillation)		
Glass fiber modified with 17-FAS [f]	~150	Grafting with fluorinated ZnO nanoparticles via chemical bath deposition	~154	1 M NaCl	60/20	12.5/11.4	Chen[18]
PVDF	119 ± 4	Grafting with fluorinated SiNPs	159 ± 2	Shale oil and gas produced water	60/20	30.2/24.8	Du[19]
PVDF	~123	Ecthing by FluoroEtch solvent, followed by silanization with FAS	~139	1M NaCl	60/20	29.2/25.3	This study
PVDF	~123	Ecthing by FluoroEtch solvent, followed by silanization with FAS	~149	1M NaCl	60/20	29.2/19.3	This study

[a] F/P: Feed solution/permeate solution; [b] SiNPs = silica nanoparticles; [c] LBL = layer-by-layer; [d] PS = polystyrene; [e] F-POSS = fluorinated-decyl polyhedraloligomeric silsesquioxane; [f] FAS = heptadecafluoro-1,1,2,2-tetrahydrodecyl trichlorosilane (or triethoxysilane).

Supplementary Note 6. Estimation of breakthrough pressure

In order to determine the water breakthrough pressure P_b of individual pores within PVDF membranes, we assume that the membrane is composed of hexagonally arranged spherical features with diameter 2*R* and pore size (i.e., inter-feature spacing) 2*D* (see Supplementary Figure 6). A force balance at the liquid-air interfaces gives:[20]

$$P_{\mathrm{b}} \approx \frac{4\pi\gamma_{\mathrm{lv}}(1-\cos\theta)}{R(2\sqrt{3}D^{*}-\pi)(\sqrt{D^{*}}-1+2\sin\theta)} \qquad (1)$$

Here, θ is the Young's contact angle, and the dimensionless parameter, $D^* = [(R+D)/R]^2$, is a measure of the air trapped underneath the liquid when it forms a composite interface with the textured surface. Using the particle diameters and pore sizes determined by analyzing the SEM images with ImageJ, the breakthrough pressure of each pore was estimated. When the breakthrough pressure of a specific pore is less than the transmembrane pressure (i.e., 1.2 kPa as measured by a low-pressure gauge), liquid water permeates into that pore.

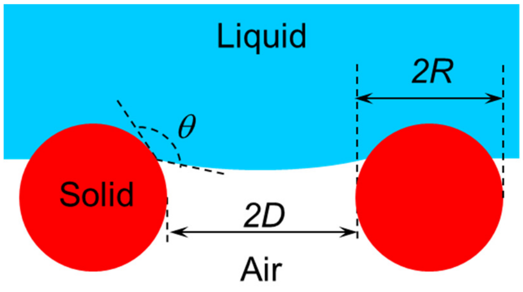

Supplementary Figure 6. Schematic depicting the membrane surface texture with spherical features.

Supplementary Note 7. Omniphobic membranes with a membrane pore size of 0.22 μm

In addition to the PVDF membranes with a membrane pore size of 0.45 μm described in the manuscript, we also fabricated omniphobic membranes using PVDF membranes with a membrane pore size of 0.22 μm (GVHP, Durapore) using the same method. The pristine membranes and the etched membranes after 5 min- and 1 h-silanization were designated as 0.22-PVDF, 0.22-PVDF-FAS-5 and 0.22-PVDF-FAS-60, respectively. The morphology of the processed PVDF membranes remained virtually unaltered compared to the pristine PVDF membranes, as evidenced by the SEM images (see Supplementary Figure 7a-7c). The omniphobic 0.22-PVDF-FAS-60 membrane possessed improved wetting resistance compared to pristine 0.22-PVDF membrane, as evidenced by the results of liquid repellency (see Supplementary Figure 7d). Further, the results (see Supplementary Figure 7e) of membrane distillation tests indicated that the 0.22-PVDF, 0.22-PVDF-FAS-5 and 0.22-PVDF-FAS-60 membranes displayed decreasing water vapor permeability with increasing wetting resistance, confirming the wetting resistance-water vapor permeability trade-off.

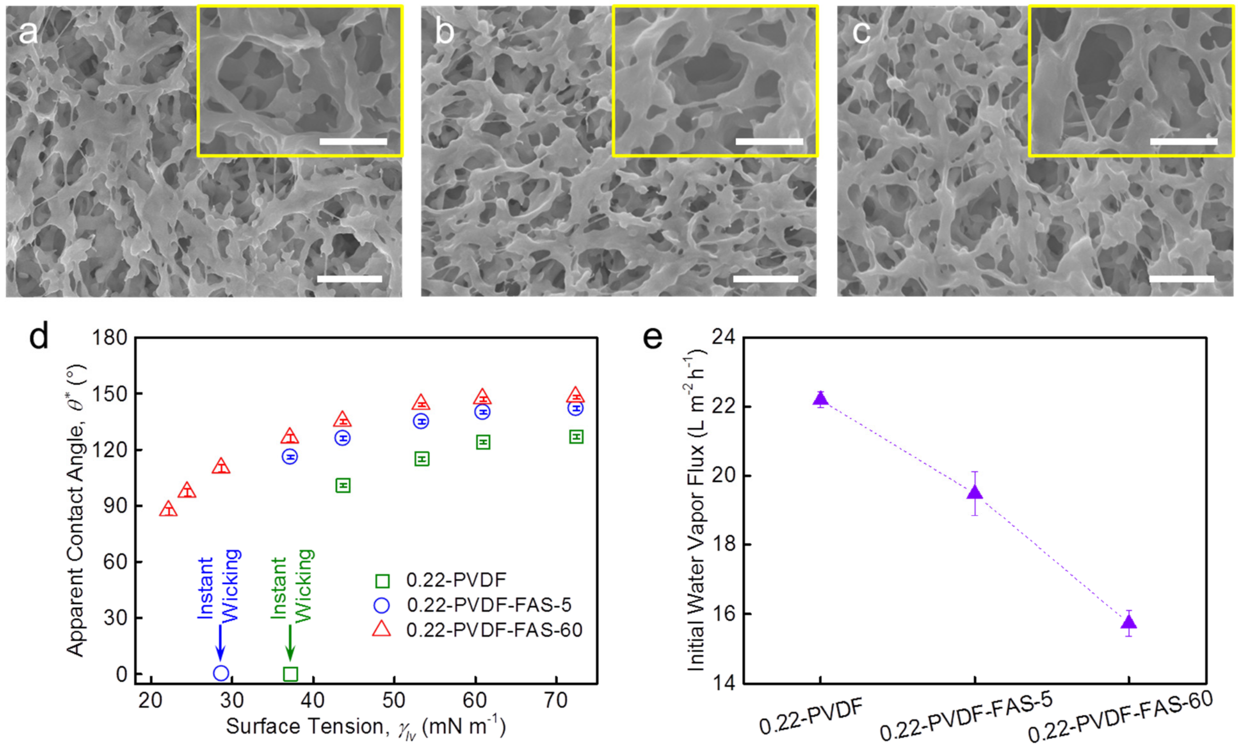

Supplementary Figure 7. Monolithic omniphobic membranes with a membrane pore size of 0.22 μm. (a-c) SEM images of (a) pristine 0.22-PVDF membrane, (b) 0.22-PVDF-FAS-5 membrane, and (c) 0.22-PVDF-FAS-60 membrane. The scale bars represent 2 μm and 1 μm (inset), respectively. (d) Apparent contact angles of liquids with different surface tensions on the membranes. (e) The initial water vapor fluxes of different MD membranes. The water vapor flux decreases (e) with increasing wetting resistance (d). Error bars represent standard deviation from three independent measurements.

Supplementary References

1 Boo, C., Lee, J. & Elimelech, M. Omniphobic polyvinylidene fluoride (PVDF) membrane for desalination of shale gas produced water by membrane distillation. *Environ. Sci. Technol.* **50,** 12275-12282, (2016).

2 Deng, L., Ye, H., Li, X., Li, P., Zhang, J., Wang, X., Zhu, M. & Hsiao, B. S. Self-roughened omniphobic coatings on nanofibrous membrane for membrane distillation. *Sep. Purif. Technol.* **206,** 14-25, (2018).

3 Tang, C. Y., Kwon, Y.-N. & Leckie, J. O. Probing the nano-and micro-scales of reverse osmosis membranes—A comprehensive characterization of physiochemical properties of uncoated and coated membranes by XPS, TEM, ATR-FTIR, and streaming potential measurements. *J. Memb. Sci.* **287,** 146-156, (2007).

4 Bielinski, A. R., Boban, M., He, Y., Kazyak, E., Lee, D. H., Wang, C., Tuteja, A. & Dasgupta, N. P. Rational design of hyperbranched nanowire systems for tunable superomniphobic surfaces enabled by atomic layer deposition. *ACS Nano* **11,** 478-489, (2016).

5 Liu, T. & Kim, C.-J. Turning a surface superrepellent even to completely wetting liquids. *Science* **346,** 1096-1100, (2014).

6 Papadopoulos, P., Mammen, L., Deng, X., Vollmer, D. & Butt, H.-J. How superhydrophobicity breaks down. *Proc. Natl. Acad. Sci. U.S.A.* **110,** 3254-3258, (2013).

7 Wang, W., Salazar, J., Vahabi, H., Joshi-Imre, A., Voit, W. E. & Kota, A. K. Metamorphic superomniphobic surfaces. *Adv. Mater.* **29,** 1700295, (2017).

8 Manickam, S. S. & McCutcheon, J. R. Characterization of polymeric nonwovens using porosimetry, porometry and X-ray computed tomography. *J. Memb. Sci.* **407,** 108-115, (2012).

9 Saffarini, R. B., Mansoor, B., Thomas, R. & Arafat, H. A. Effect of temperature-dependent microstructure evolution on pore wetting in PTFE membranes under membrane distillation conditions. *J. Memb. Sci.* **429,** 282-294, (2013).

10 Islam, M. A. & Ulbricht, M. Microfiltration membrane characterization by gas-liquid displacement porometry: Matching experimental pore number distribution with liquid permeability and bulk porosity. *J. Memb. Sci.* **569,** 104-116, (2019).

11 Huang, Y. X., Wang, Z. X., Jin, J. & Lin, S. H. Novel janus membrane for membrane distillation with simultaneous fouling and wetting resistance. *Environ. Sci. Technol.* **51,** 13304-13310, (2017).

12 Lee, J., Boo, C., Ryu, W. H., Taylor, A. D. & Elimelech, M. Development of omniphobic desalination membranes using a charged electrospun nanofiber scaffold. *ACS Appl. Mater. Interfaces* **8,** 11154-11161, (2016).

13 Woo, Y. C., Kim, Y., Yao, M., Tijing, L. D., Choi, J. S., Lee, S., Kim, S. H. & Shon, H. K. Hierarchical composite membranes with robust omniphobic surface using layer-by-layer assembly technique. *Environ. Sci. Technol.* **52,** 2186-2196, (2018).

14 Zheng, R., Chen, Y., Wang, J., Song, J. F., Li, X. M. & He, T. Preparation of omniphobic PVDF membrane with hierarchical structure for treating saline oily wastewater using direct contact membrane distillation. *J. Memb. Sci.* **555,** 197-205, (2018).

15 Lu, X. M., Peng, Y. L., Qiu, H. R., Liu, X. R. & Ge, L. Anti-fouling membranes by manipulating surface wettability and their anti-fouling mechanism. *Desalination* **413,** 127-135, (2017).

16 Lu, C., Su, C., Cao, H., Ma, X., Duan, F., Chang, J. & Li, Y. F-POSS based omniphobic membrane for robust membrane distillation. *Mater. Lett.* **228,** 85, (2018).

17 Lu, K. J., Zuo, J., Chang, J., Kuan, H. N. & Chung, T. S. Omniphobic hollow-fiber membranes for vacuum membrane distillation. *Environ. Sci. Technol.* **52,** 4472-4480, (2018).

18 Chen, L. H., Huang, A., Chen, Y. R., Chen, C. H., Hsu, C. C., Tsai, F. Y. & Tung, K. L. Omniphobic membranes for direct contact membrane distillation: Effective deposition of zinc oxide nanoparticles. *Desalination* **428,** 255-263, (2018).

19 Du, X., Zhang, Z., Carlson, K., Lee, J. & Tong, T. Z. Membrane fouling and reusability in membrane distillation of shale oil and gas produced water: Effects of membrane surface wettability. *J Membrane Sci* **567,** 199-208, (2018).

20 Kota, A. K., Li, Y., Mabry, J. M. & Tuteja, A. Hierarchically structured superoleophobic surfaces with ultralow contact angle hysteresis. *Adv. Mater.* **24,** 5838-5843, (2012).
