# Supplementary material for: Trade-off in membrane distillation with monolithic omniphobic membranes
Source: Nat Commun. 2019 Jul 19;10:3220. doi: 10.1038/s41467-019-11209-6 (PMC6642111; doi:10.1038/s41467-019-11209-6)
Supplement: Supplementary file 2 — Description of Additional Supplementary Files [file 41467_2019_11209_MOESM2_ESM.pdf]

Description of Additional Supplementary Files

File name: Supplementary Movie 1

Description: This video illustrates the immersion of pristine PVDF membrane in the etchant for ~1 s.

File name: Supplementary Movie 2

Description: This video (32X speed) illustrates the different wetting resistance of pristine and processed PVDF membranes subjected to a 12-cm column of water + 30% ethanol. The membranes were sandwiched between two vertical glass tubes. The water + 30% ethanol completely permeated through the pristine PVDF membrane. The scale bar represents 1 cm.

File name: Supplementary Movie 3

Description: This video (32X speed) illustrates the different wetting resistance of pristine and processed PVDF membranes subjected to a 12-cm column of 100% ethanol. The membranes were sandwiched between two vertical glass tubes. The 100% ethanol completely permeated through the pristine PVDF membrane and PVDF-FAS-5 membrane. The scale bar represents 1 cm.

File name: Supplementary Movie 4

Description: This video illustrates the dynamic formation of a non-wetted pore on an omniphobic membrane.

File name: Supplementary Movie 5

Description: This video illustrates the dynamic formation of a wetted pore on a hydrophobic membrane.

File name: Supplementary Movie 6

Description: This video illustrates the dynamic formation of 5 wetted pores (water permeating through the first layer of membrane and contacting the second layer) on a hydrophobic membrane consisting of several pores with different sizes.

File name: Supplementary Movie 7

Description: This video illustrates the dynamic formation of 2 wetted pores (water permeating through the first layer of membrane and contacting the second layer) on an omniphobic membrane with the same geometry as the hydrophobic membrane shown in Supplementary movie 6.
